# A conserved motif in the immune-subdominant RAP-1 related antigen of *Babesia bovis* contains a B-cell epitope recognized by antibodies from protected cattle

**DOI:** 10.3389/fimmu.2024.1380660

**Published:** 2024-04-24

**Authors:** Manuel J. Rojas, Reginaldo G. Bastos, Jinna Navas, Jacob M. Laughery, Paul A. Lacy, Carlos E. Suarez

**Affiliations:** ^1^ Department of Veterinary Microbiology & Pathology, College of Veterinary Medicine, Washington State University, Pullman, WA, United States; ^2^ Animal Health Department, Universidad Nacional de Colombia, Bogotá, Colombia; ^3^ Animal Disease Research Unit, Agricultural Research Service, United States Department of Agriculture, Pullman, WA, United States

**Keywords:** RRA: RAP-1 related antigen, RAP-1: rhoptry associated protein-1 *Babesia bovis*, babesiosis, epitopes, vaccines, RAP-1, RRA

## Abstract

**Introduction:**

*Babesia bovis*, a tick-borne apicomplexan parasite causing bovine babesiosis, remains a significant threat worldwide, and improved and practical vaccines are needed. Previous studies defined the members of the rhoptry associated protein-1 (RAP-1), and the neutralization-sensitive rhoptry associated protein-1 related antigen (RRA) superfamily in *B. bovis*, as strong candidates for the development of subunit vaccines. Both RAP-1 and RRA share conservation of a group of 4 cysteines and amino acids motifs at the amino terminal end (NT) of these proteins.

**Methods and results:**

Sequence comparisons among the RRA sequences of several *B. bovis* strains and other *Babesia* spp parasites indicate a high level of conservation of a 15-amino acid (15-mer) motif located at the NT of the protein. BlastP searches indicate that the 15-mer motif is also present in adenylate cyclase, dynein, and other ATP binding proteins. AlphaFold2 structure predictions suggest partial exposure of the 15-mer on the surface of RRA of three distinct *Babesia* species. Antibodies in protected cattle recognize a synthetic peptide representing the 15-mer motif sequence in iELISA, and rabbit antibodies against the 15-mer react with the surface of free merozoites in immunofluorescence.

**Discussion and conclusion:**

The presence of the 15-mer-like regions in dynein and ATP-binding proteins provides a rationale for investigating possible functional roles for RRA. The demonstrated presence of a surface exposed B-cell epitope in the 15-mer motif of the *B. bovis* RRA, which is recognized by sera from protected bovines, supports its inclusion in future subunit epitope-based vaccines against *B. bovis*.

## 1 Introduction

Bovine babesiosis is an acute hemolytic and persistent infectious disease of worldwide impact that is mainly caused by the tick-borne apicomplexan parasites *Babesia bovis*, *B. bigemina*, and *B. divergens*, and remains an important threat to cattle production globally. Currently, the disease can be controlled, although inefficiently, using tick control measures, babesicidal drugs, and live vaccines (1, 2). Since all these methods have severe limitations (1, 2), new control approaches, including subunit vaccines, are urgently needed.

*Babesia* parasites undergo sexual reproduction in the tick midgut, are transmitted transovarially, and can only infect erythrocytes in their vertebrate hosts (1, 2). Like other apicomplexans, *Babesia* parasites contain apical end organelles that include the rhoptries, micronemes and spherical bodies, among other conserved structures (3, 4). *Babesia* sporozoites, which are inoculated with the saliva of the tick vectors, are responsible for the initial step of infection of erythrocytes in the vertebrate host. Other life stages in the vertebrate hosts include the ring shaped trophozoites, merozoites (the invasive stage), and pre-sexual stage forms, which can develop in fully mature sexual forms upon ingestion by competent ticks (5). Invasion by merozoites involves initial recognition of the target erythrocyte by molecules located on the parasite surface, followed by re-orientation and apposition of the apical end with the erythrocyte surface membrane, and the sequential secretion by the organelles of the apical complex, including the micronemes and the rhoptries (3, 4). The rhoptries are a pair of relatively large, club-shaped organelles containing proteins that are secreted during invasion. The parasites then actively enter erythrocytes by a mechanism mediated by its actin-myosin motor (6). Upon entry, the parasite is surrounded by a transient parasitophorous membrane that disappears shortly after invasion (7). It has been postulated that rhoptry proteins are involved in the formation, and perhaps also subsequent dissolution, of the parasitophorous membrane, but they may play several roles and are important to establish and controlling the target cell infection (3, 4). Because of their important roles and significance, rhoptry proteins, and other apical complex proteins are considered prime vaccine candidates (1, 2, 8, 9).

Interestingly, all Piroplasmid organisms sequenced so far contain variable copies of genes encoding for the rhoptry associated protein-1 (RAP-1) family, which appear to be unique to this order (10). Piroplasmid RAP-1 members are distinct to the Plasmodial RAP-1 (10), and they all possess characteristic sequence features that include signal peptides and presence of the RAP-1 domain, that consists of a ~300 amino acid long sequence with 4 conserved cysteine residues and a well-conserved 14 or 15 amino acid sequence (15-mer), in addition to other shorter amino acid motifs (11–13). While *Babesia* RAP-1 proteins contain just a single ~310 amino acid long “RAP-1 domain” located in the amino terminal (NT) portion of the molecule (10), the *Theileria* RAP-1 proteins typically contain more than a single RAP-1 domain organized in tandem. The carboxyl end segment (CT) of *Babesia* RAP-1s is usually not conserved among different parasites, and it may contain repeated motifs, as is the case of the *B. bovis* RAP-1 (12).

Antigenic characterization of *B. bovis* RAP-1 molecules showed that while the NT region contains at least 2 defined T-cell epitopes (14) and at least one monoclonal antibody-defined B-cell epitope (12), the CT terminus has at least a single T-cell epitope and at least two monoclonal antibody-defined B-cell epitopes (12). However, neither the monoclonal antibody defined B-cell nor the T-cell epitopes in the NT region include the most species-conserved regions of the molecule (between aa 70-130) (12, 14). Overall, the piroplasmid RAP-1 (pRAP-1) molecules are highly antigenic and have been widely used for the development of *Babesia* serological diagnostic tests (15, 16). However, most anti-RAP-1 antibodies developed during infections are reactive with B-cell epitopes located in the CT region of the molecule, strongly suggesting that the non-species conserved CT portions of RAP-1 are highly antigenic (12). Remarkably, the conserved Babesia RAP-1 NT regions, which likely contain its functional domains, are also less immunogenic, or sub- immunodominant in terms of antibody induction and reactivity, compared with the sequences in the less inter-species conserved CT-regions (12, 17).

Nevertheless, despite intensive bioinformatic, molecular, and immunological characterization, the specific function(s) of the pRAP-1 molecules remains unknown. In addition, several studies found that antibodies against RAP-1 can partially inhibit invasion of erythrocytes by *B. bovis* sporozoites and merozoites (18), suggesting a role in invasion. Therefore, RAP-1 antigens were included in several vaccine formulations that showed some degree of protection (9). Nevertheless, neither full size RAP-1 nor a truncated version of RAP-1 including its conserved NT region, were able to confer protection against challenge with virulent strains of *B. bovis* when used as single vaccine components (19).

Due in part to their merozoite surface exposure, relatively high level of expression, and antigenicity, the canonical pRAP-1 molecules were identified relatively early in Babesia research leading to novel serological diagnostic methods and test of experimental subunit vaccines (9, 12, 14–16, 19, 20). However, it was not until the full sequencing of the *B. bovis* genome was achieved, that another RAP-1 like protein, the RAP-1 Related Antigen (RRA) was discovered (21, 22). RRA is a truncated version of RAP-1, including the well-conserved NT portion with the RAP-1 domain, but lacking a CT end containing antigenic degenerate repeats. In contrast to RAP-1, RRA is poorly antigenic, and deemed as an immune-subdominant antigen, which is expressed, at least in blood stages of the parasite, at much lower levels than the canonical RAP-1 (22). Most significantly, antibodies against RRA can block, at least in part, invasion of the erythrocyte by the parasites (22). Remarkably, while all sequenced Babesia parasites appear to contain a single copy *rra* gene, this gene is not present in other piroplasmid parasites, suggesting that the *rra* gene evolved after speciation of Babesia parasites. Thus, the presence of a *rra* gene might be considered a defining feature and a marker of *sensu stricto* Babesia parasites (10).

In this study we address conservation, function, structural context, and immunogenic characteristics of the conserved 15-mer amino acid motif of RRA, which is highly related and overlaps in sequence with a previously characterized and conserved 14-mer motif characteristic of the canonical pRAP-1 sequences, as a possible candidate for inclusion in future epitope-based subunit vaccines against *B. bovis*. To determine whether the conserved 15-mer motif includes B-cell epitopes that are recognized by antibodies in protected cattle, we analyzed IgM and IgG circulating levels in cattle that were previously vaccinated with an attenuated strain of *B. bovis*, and further challenged with virulent parasites (10).

The data gathered in this study confirmed a high level of conservation for the 15-mer motif among *B. bovis* strains, in other Babesia species, and in otherwise unrelated proteins in databases. We also describe the presence of a sub-immunodominant surface exposed B-cell epitope in the conserved 15-mer motif of RRA, which is recognized by antibodies in calves protected against challenge with virulent parasites.

## 2 Materials and methods

### 2.1 Bioinformatics and in silico structural analysis

The *B. bovis*, *B. bigemina*, and *B. ovata* RRA sequences (XP_001610950.1, XP_012766682.1, XP_028866572.1, respectively) were modeled with AlphaFold Data (2022) DeepMind Technologies Limited (23, 24). In addition, molecular graphics and analyses were performed with UCSF ChimeraX, developed by the Resource for Biocomputing, Visualization, and Informatics at the University of California, San Francisco (25).

Database sequence searches homologies were performed using BlastP (Protein BLAST: search protein databases using a protein query (nih.gov)). Multiple sequence alignments were performed using Clustal Omega (Clustal Omega < Multiple Sequence Alignment < EMBL-EBI)

Partial size *rra* genes from DNA extracted the *B. bovis* Mo7 (26), S79-T3BO (27), Argentina (28) RRA and Australia strains (29) were amplified using primers: RRA-F (5’ GGGCAATGTGCTATCTTGGATACTG 3’) and RRA-Rev (5’ CAAACTCTGTCGTAAAGGTGCCCG 3’):. All amplicons were cloned into pCR-TOPO 2.1 vector (Invitrogen, CA, USA) and sequenced in full by Eurofins MWG Operon (Louisville, KY).

### 2.2 Parasites and sera from *B. bovis* protected cattle

The Mo7 biological clone of *B. bovis* (20) was used as a source of antigen for immunoblots and immunofluorescence analysis. Parasites were grown in long term microaerophilous stationary-phase culture by previously described methods (20).

Pre-immune and immune sera from the *B. bovis* experimentally infected cattle used in the ELISA analysis were generated in a previous study (30, 31).

### 2.3 Synthetic peptides, recombinant RRA, and polyclonal antibody production

RRA synthetic peptides were obtained from Pacific Immunology Corp. (Ramona, CA). The sequence of the peptides used in this study is as follows: RRA NT: PTIRLPNTYQLEAAF (>80% purity as determined by HPLC analysis); RRA CT: PVKWAVNLIENDDAPYGW (>80% purity as determined by HPLC analysis); The *B. bovis* derived CT-peptide representing a B-cell epitope of RAP-1 (12) was produced by Vivitide (Louisville, KY, USA): PTKEFFREAPQATKHFLDENIGA (>95 purity as determined by HPLC analysis). Rabbit antibodies were generated against the peptide 15-mer RRA-NT *B. bovis*: PTIRLPNTYQLEAAF; The peptide 15-mer RRA NT was conjugated to keyhole limpet hemocyanin (KLH) and used in the immunization of rabbits by Pacific Immunology Corp. (Ramona, CA). The specificity of the rabbit antibody against the 15mer peptide was also tested by iELISA using synthetic 15mer and control peptides coated into ELISA plates as described in section 2.4, below. The specificity of the monoclonal antibodies reactive against the *B. bovis* RAP-1 CT Babb75 was previously described (12).

The full size *B. bovis* RRA gene (XP_001610950.1) and RAP-1NT (12, 14) sequences were codon optimized for expression in prokaryotic cells, synthesized, and cloned into vector pET-30a(+) with 6His tag for protein expression in *E. coli* BL21 star (DE3) cells. After expression, the proteins were obtained from inclusion bodies and purified by Ni-column with a purification rate of >85%. The starting volume was 1 L of Terrific medium broth, with a final yield of 18.13 mg. Level of protein purity and expected molecular weight were determined by SDS–PAGE and Western blot. Briefly, the proteins were separated in 4–20% Precast TGX gel (BioRad, Hercules, CA, USA) and stained with Coomassie blue using a standard protocol (**Supplementary Figure 1**). For Western blot analysis, the SDS–PAGE separated proteins were transferred to a PVDF membrane using manual transfer and probed with HRP-conjugated His-Tag Monoclonal antibody (Proteintech, Rosemont, IL, USA). The size marker used in SDS–PAGE and Western blot was Prestained Precision Plus Protein Dual Color Standard (BioRad, Hercules, CA, USA). Identity confirmation of rRRA protein was performed by LC–MS/MS, as per the third-party company’s protocol (GenScript, Piscataway, NJ, USA). The recombinant protein was stored at −80 °C in a 50 mM Tris-HCl, 150 mM NaCl, 10% Glycerol, 0.2% SDS, pH 8.0 buffer until further use.

### 2.4 ELISA and immunoblots

Indirect ELISA (iELISA) for synthetic peptides were performed on 96-well Immulon™ 2HB microtiter plates (Thermo Fisher Scientific, Waltham, MA) coated overnight at 4°C with 50 μl of synthetic peptides RAP-1 CT, RAP-1 NT, RRA NT, RRA CT, and randomized RRA-NT peptide (sequence: DQTAINREPYPTALL) (2 μg/ml). Plates were then washed three times using 200 μl blocking buffer (0.2% I-Block™ in 1xPBS with 0.1% Tween 20) and blocked with 300 μl of the same buffer for one hour at room temperature. After blocking, primary antibody was diluted 1:10 for IgG, or 1:20 for IgM detection, and 50 μl was added to have technical duplicate wells. For specificity of the rabbit antibody against the RRA NT 15mer peptide, the primary immune and pre-immune rabbit antibodies were diluted 1:500 in blocking buffer. Plates were incubated for one hour at room T° and then washed five times in 200 μl blocking buffer. Then, 50 μl of a 1:500 dilution of anti-bovine total IgG or 1:300 dilution of anti-bovine total IgM peroxidase labeled secondary antibodies (SeraCare, Milford, MA) were added to each well, and plates were incubated for 45 minutes at room T°. For the plates incubated with rabbit primary antibodies, the secondary goat labeled secondary antibodies anti-rabbit IgG was diluted 1:1000 in identical buffer. After incubation, plates were washed four times using 200 μl ELISA washing buffer and two times with 200 μl PBST buffer. Following, 55 μl of SureBlue™ TMB (SeraCare, Milford, MA) was added to each well and plates were incubated for 10 minutes. After addition of 55 μl TMB stop solution (SeraCare, Milford, MA), absorbance was measured at 450 nm using the SpectraMax^®^ 190 plate reader (Molecular Devices, San Jose, CA). The serum samples analyzed hereby were generated in a previous study (30, 31) involving vaccination of cattle with the *in vitro* culture attenuated strain of *B. bovis* Att- S74-T3Bo *B. bovis* that was challenged with a virulent strain of the parasite Vir-S74-T3Bo *B. bovis*. Statistical significance was determined using a paired 2 tails type 1, T-test. The previous animal study was reviewed and approved by Washington State University Institutional Animal Care and Use Committee (IACUC protocol number 2020-51).

### 2.5 Immunoblot analysis

**Figure 1:**
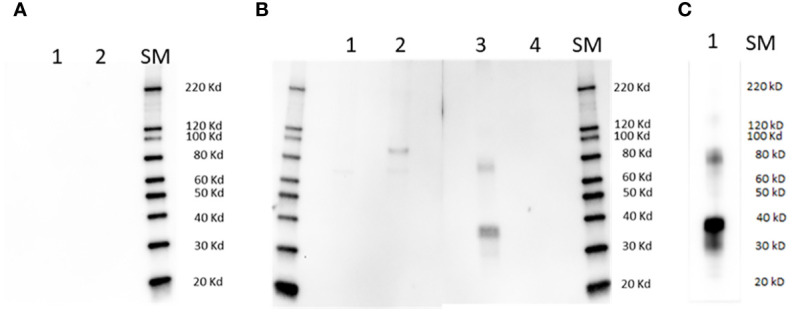
Immunoblot analysis. Lanes in **(A)** are as follows: 1] full size recombinant RRA; 2] Unrelated recombinant control protein; lanes in **(B)** are: 1] non-infected bovine erythrocyte lysate; 2] lysate of (*B*) *bovis* infected bovine erythrocytes; 3] full size recombinant RRA; 4] recombinant control protein, (*B*) *bovis* rRAP-1. Membranes represented in **(A, B)** were incubated with pre-immune and Immune rabbit anti RRA - sera respectively. **(C)**: Lane 1: full size recombinant RRA, incubated with monoclonal anti HIS antibody as the primary antibody. SM, Size markers.

Briefly, total antigens (2 μg/lane) from uninfected bovine RBC or Mo7 iRBC (2% percentage of parasitized erythrocytes, PPE) were ran through 4-20% TGX™ gels (Bio-Rad Laboratories, Hercules, CA), using 5X Sample Buffer containing 10% 2-Mercaptoethanol (GenScript, Piscataway, NJ, USA). Antigens were then transferred to nitrocellulose membranes using iBlot 2™ (Invitrogen, Waltham, MA), and the membranes were blocked 1 hour in 5% milk. Membranes were then washed one time in 1xPBS with 0.1% Tween 20 (PBS-T) and incubated with a 1:500 dilution of either pre-immune rabbit sera or rabbit anti 15-mer antibody serum in 5% milk for one hour rocking at room temperature. After that, membranes were washed three times using PBS-T and incubated with a 1:1000 dilution of anti-rabbit IgG peroxidase labeled secondary antibodies (SeraCare, Milford, MA) in 5% milk for 30 minutes rocking at room T°. Membranes were then washed again three times in PBS-T and incubated with Prometheus Protein Biology Products 20-300B ProSignal^®^ Pico ECL Reagent (company)?. Visualization of immune complexes was performed using the Azure™ Imaging System (Azure Biosystems, Dublin, CA). The immunoblot in **Figure 1C** was performed using 1:500 anti-His tag antibody (Bio-Rad Laboratories, Hercules, CA), followed by peroxidase labeled anti-Mouse antibody 1:5000 (SeraCare, Milford, MA).

### 2.6 Immunofluorescence

Extraerythrocytic free merozoites were isolated from cultured *B. bovis* Mo7 parasites using centrifugation, as previously described (32). The samples were then washed in 3% bovine serum albumin (BSA) and aliquoted for use in cell permeabilized and non-permeabilized indirect immunofluorescence assays (IFA). For permeabilized IFA, samples were first smeared on a slide, fixed for 5 min in 100% acetone, and then incubated with 0.1% Triton X-100. The slides were then incubated in 10% BSA for 1 h with a combination of anti-RAP-1 antibodies (2 ug/mL) and anti-RRA peptide antibodies (1/500) (31). The slides were washed three times with PBS and incubated in 10% BSA with goat anti-rabbit IgG Alexa Fluor^®^ 555 (red fluorescence) and goat anti-mouse IgG Alexa Fluor^®^ 488 (green fluorescence) (Thermo Fisher Scientific, Waltham, MA). The slides were then washed three times with PBS and mounted with a drop of Prolong™ Gold Anti-fade with 4′,6-diamidino-2-phenylindole (DAPI) (Thermo Fisher, Waltham, MA, USA) and cover slip. Non-permeabilized samples were incubated with the antibodies, washed within a 1.5 mL tube, then fixed on a slide, and mounted in an identical manner as the permeabilized samples. The slides were analyzed using a Leica SP8-X White Light Laser point scanning confocal microscope (Leica Microsystems, Wetzlar, Germany). The digital images were processed using Leica LAS X analysis software (Leica Microsystems, Wetzlar, Germany) to produce individual and merged images.

## 3 Results

### 3.1 In silico analysis of RRA and RRA conserved motifs

**Figure 2:**
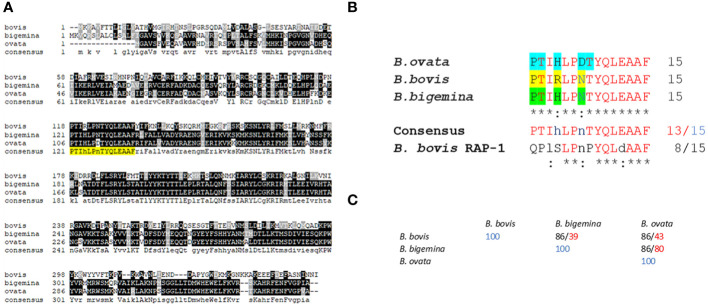
Sequence alignments and bioinformatics analysis: **(A)** Multiple sequence alignment of predicted RRA amino acid sequences of *Babesia bovis* (XP_001610950.1); (*B*) *bigemina* (XP_012766682.1), and (*B*) *ovata* (XP_028866572.1). The sequence of the conserved 15-mer motif is highlighted in yellow. **(B)** Detailed sequence alignment of the 15-mer motifs of (*B*) *bovis*, (*B*) *bigemina*, and (*B*) *ovata*. The residues predicted to be exposed on the surface of the molecule by AlphaFold are highlighted in blue, yellow and green. **(C)** Percentage of identity among the 15- mer sequences of (*B*) *bovis*, (*B*) *bigemina*, and (*B*) *ovata* (black font). The numbers in red font correspond to overall sequence identity among the full size RRA proteins.

Sequence comparisons among the RRA proteins from *B. bovis*, *B. bigemina*, and *B. ovata*, revealed strong conservation of the 15-mer sequence that overlaps and extends in one residue the 14-mer motif previously identified as conserved among canonical RAP-1 proteins (11, 13) (**Figures 2A, B**).

A detailed comparison of the 15-mer conserved motif sequences among the RRAs from *B. bovis*, *B. bigemina*, *B. ovata*, and *B. bovis* RAP-1, is shown in **Figure 2B**. At least 13 out of the 15 amino acids in this sequence are strictly conserved among the RRA sequences from these three distinct selected *Babesia* species, and 8 residues are also strictly conserved in *B. bovis* RAP-1 (**Figures 2B, C**). Furthermore, the two variable residues in these sequence comparisons (HxR; DxN) are conservative substitutions (33). In addition, and in all cases, the identities among the RRA 15-mer motifs among these three parasites are significantly higher than the identities among their RRA full size amino acid sequences (**Figure 2C**), suggesting that the region including the conserved motif has a lower evolutionary rate than other regions in the RRA gene. However, RRA, including the 15-mer motif, is fully conserved among *B. bovis* strains from Argentina, Mexico, Australia, as well as in the clonal Mo7 strain (**Supplementary Figure 1**). Also, as previously observed (26), the 15-mer motif is also well conserved among RRA and RAP-1 of *B. bovis* (**Supplementary Figure 2**).

Interestingly, BlastP database searches using just the conserved 15 amino acid motif region as a query revealed strong similarity with sequences present in at least two distinct adenylate cyclase type 10-like proteins (GenBank: KMQ94175 and XP_029155514.1) (red boxes, **Supplementary Figures 3**, **4**), as well as partially conserved in other ATP-binding proteins, such as dynein (ie: GenBank XP_019924815), an ATP-binding cytoskeletal motor protein. An alignment of the full *B. bovis* RRA amino acid sequence with the sequence of the adenylate cyclase containing homology to the 15-mer motif (AC1) using CLUSTALW revealed overall 22.7% of identity among RRA and AC1 (**Supplementary Figures 3**, **4**). However, the highest similarity among these two proteins is present in the region representing the 15-mer motif (overall 67% identity, with 2 conservative or semiconservative substitutions in a 13 amino acid stretch, as shown in **Supplementary Figure 3**). Furthermore, the sequence homology extends along both NT and CT ends of RRA and, significantly, includes also one of the highly conserved cysteine residues (**Supplementary Figures 3**, **4**). Consistently, BlastP database searches using just the 13 amino acid regions from AC that almost fully matches the 15-mer conserved RRA sequence as a query (**Supplementary Figure 3**), produced significant hits with the RRA of *B. bovis* and *B. bigemina*, and other ATP-Binding (ABC) proteins (data not shown).

**Figure 3:**
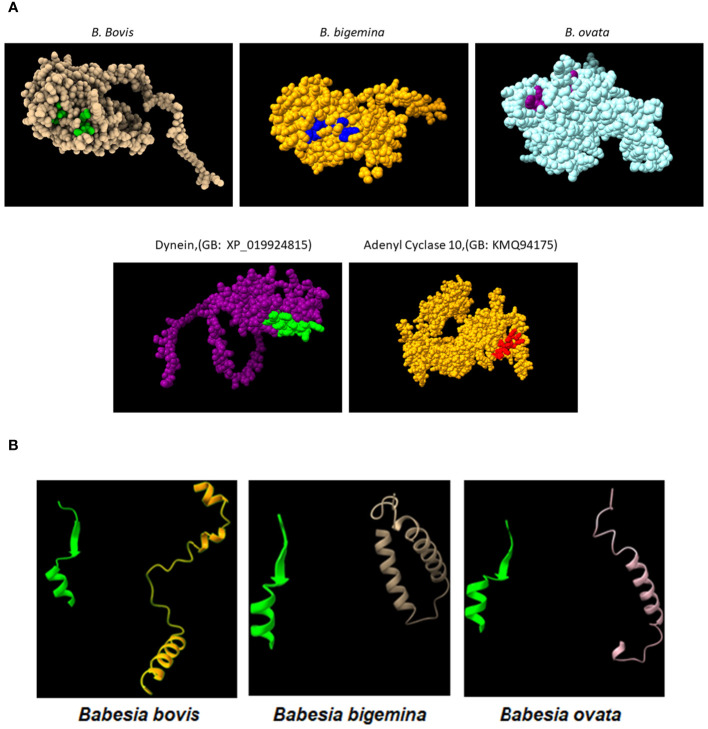
**(A)** Upper panel: AlphaFold generated surface structure predictions of the RRA derived from (*B*) *bovis*, (*B*) *bigemina*, and (*B*) *ovata*. Residues in green, blue and purple color correspond to surface exposed residues of the 15-mer motif of RRA. Lower panel: AlphaFold generated surface structure predictions of Dynein (GenBank access #: XP_019924815), and Adenyl Cyclase 10 (GenBank access #:KMQ94175). The areas marked in green and red correspond to surface exposed sequences of the conserved 15mer sequences in these two proteins. **(B)** Conserved Alphafold predicted structural features of the 15-mer motif in the (*B*) *bovis*, (*B*) *bigemina*, and (*B*) *ovata* RRA proteins (green) compared with their structurally distinct CT regions (non-yellow colors).

In addition, comparisons of AlphaFold predicted surface structures of RRA from *B. bovis*, *B. bigemina*, and *B. ovata* (**Figure 3A**), suggest overall structural conservation among RRA proteins despite their amino acid sequence differences (**Figure 2A**). Interestingly, the region including the 15-mer motif in RRA of *B. bovis*, *B. bigemina* and *B. ovata*, have identical predicted structures, consisting of α-helix and β-sheet regions joined by a non-structured linker (**Figure 3B**). The 15-mer motif of RRA described hereby is a one residue extension version of the 14-mer motif in RAP-1 proteins (34) which contains its more conserved residues buried in the core of the protein, which overall has a globin-like structure. Remarkably, this includes the key L-122 and P-123 residues of the 15-mer motif that are conserved on other pRAP-1 superfamily structural homologues identified in more distant species such as Plasmodium and Toxoplasma (34). However, residues in the relative positions 1, 2, 4, and 7 (differentially colored in **Figure 3A** and highlighted in **Figure 2B**) of the 15-mer conserved region are predicted to be exposed on the surface of the RRAs derived from the 3 parasites compared hereby. Thus, according to these structural AlphaFold models, at least some residues of the conserved 15-mer of the RRA molecules might be exposed on the surface of the molecules (**Figure 3B**). According to this prediction, there are four exposed residues in matching positions for the RRAs of the three parasites (**Figure 2A** and highlighted residues in **Figure 2B**), except for *B. ovata*, which has an additional predicted surface exposed residue (T-113). While the matching 15-mer surface exposed predicted residues P and T are fully conserved among the three molecules (**Figures 2A, B**), the other two matching residues (Positions 4 and 7 in **Figure 2B**) are the only ones which are variable among the three RRA molecules compared in this study. Interestingly, two out of these four predicted surface exposed amino acid residues (#4 and #7 in **Figure 2B**) are the only ones that vary in the 15-mer motif sequences among these three RRA molecules. Notably, as pointed out before, the amino acid changes at these two positions can be considered conservative substitutions (33) which do not likely alter the structural features of this region and may have no significant influence on the functional role of the motif. In contrast to the NT regions, and consistent with previous findings (34), the CT end regions of these three RRA proteins do not show AlphaFold-predicted species-conserved structures at all (**Figures 3A, B**). Interestingly, AlphaFold surface structure predictions for the Adenyl-Cyclase and Dynein molecules which also contain versions of the 15-mer, as described above, also show globular shapes which appear to be similar to the RRAs, with residues of their 15-mer-like sequences also exposed on their surfaces (**Figure 3A**).

Altogether, the data implies that the conserved 15-mer motif may be critical for RRA, as well as for RAP-1, functions and suggests that it may have surface exposed residues that might be the target of invasion-neutralizing antibodies.

### 3.2 The conserved 15-mer includes a partially surface exposed B-cell epitope recognized by *B. bovis* protected cattle

To analyze immunogenicity and confirm exposure conserved motif, we first generated rabbit polyclonal antibodies against a peptide representing the 15-mer motif of the *B. bovis* RRA. These antibodies were used in ELISA, immunoblots and immunofluorescence analysis of infected erythrocytes and extraerythrocytic merozoites using permeabilized and non-permeabilized *B. bovis* parasites. In addition, we also examined whether antibodies in *B. bovis* protected calves recognized B-cell epitopes that might be in the *B. bovis* 15-mer conserved motif.

The sera from a rabbit immunized with the 15-mer motif of RRA, but not pre-immune rabbit sera, strongly reacted with a synthetic peptide representing the 15-mer motif of RRA, but not with a randomized version of the same peptide in an ELISA test (**Supplementary Figure 5A**), confirming its specificity. In addition, the rabbit antibodies against the 15-mer of RRA also reacted with recombinant RRA antigen (rRRA) generating a ~38 kDa band which matches the predicted size of this recombinant protein, but do not react with rRAP-1N, a recombinant antigen that represents the conserved region of *B. bovis* RAP-1 that includes the 15-mer sequence described in **Figure 2B** (**Figure 1**, **Supplementary Figure 5B**). However, as shown in the Figures, the rabbit antibodies against the 15-mer of RRA also recognized a HIS-containing ~78 kD recombinant protein that was co-purified with rRRA. This pattern of reactivity was similar to the one obtained using anti-HIS antibodies as the primary antibody in immunoblots (**Figure 1C**, **Supplementary Figure 5C**), which include reactivity with a ~78 kDa protein, suggesting that the larger recombinant antigen may represent a RRA dimer. The rabbit antibodies against the 15-mer motif of RRA only recognized a native ~78 kDa *B. bovis* protein, in a lysate of *B. bovis* infected erythrocytes derived from *in vitro* cultured parasites in immunoblot analysis (**Figure 1B**). Consistent with what was found for rRRA, this pattern of reactivity suggests that the native ~78 kDa protein recognized by the rabbit anti15-mer RRA antibodies may represent a dimer of native RRA. Alternatively, it is possible that the amounts of monomeric RRA, but not of the dimeric form, present in the culture lysates, is below the limit of detection of the immunoblot method as it was suggested in previously published studies (22). It is also possible that the larger ~80 kDa band represents post-translationally modified native RRA, or a complex of RRA with an unknown molecule. No reactivities were detected for the rabbit anti-15-mer antibodies against proteins in a non-infected bovine erythrocyte lysate, and no antigens in the lysates of infected and non-infected erythrocytes were recognized by rabbit pre-immune sera (**Figures 1A, B**).

**Figure 4:**
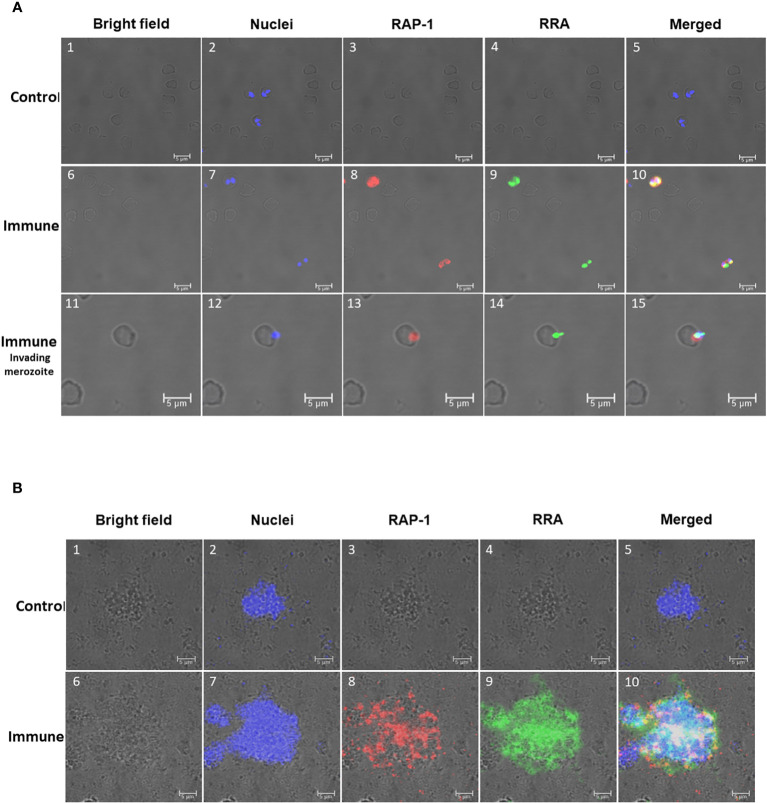
Immunofluorescence analysis of: **(A)** Permeabilized *in vitro* cultured (*B*) *bovis* infected bovine erythrocytes. Control Panels 1-5 were incubated with pre-immune rabbit and Tryp control mAb. Immune Panels 6-15: incubated with monoclonal antibody BABB75, reactive with an epitope in (*B*) *bovis* RAP-1 and rabbit antisera against 14mer RRA. RAP-1: Alexa fluor anti-mouse 555 in the red channels; RRA: Alexa fluor anti rabbit 488 in the green channels. Bright field, nuclei, RAP-1, RRA and Merged images as indicated in the Figure. Nuclei staining: DAPI in the blue channels. Immune invading merozoite: selected image showing a merozoite invading an erythrocyte stained as indicated above. **(B)** Non-permeabilized extracellular *in vitro* cultured (*B*) *bovis* merozoites: Control Panels 1-5: incubated with pre-immune rabbit and Tryp control mAb. Panels 6-10 (Immune) incubated with monoclonal antibody BABB75, reactive with an epitope in (*B*) *bovis* RAP-1and rabbit antisera against 14mer RRA. RAP-1: Alexa fluor anti-mouse 555 in the red channels; RRA: Alexa fluor anti rabbit 488 in the green channels. Bright field, nuclei, RAP-1, RRA and Merged images as indicated in the Figure. Nuclei staining: Hoechst 33347 in the blue channels.

In addition, we compared the pattern of immunofluorescence reactivity of the anti-15-mer motif rabbit antibody (green fluorescence) with the monoclonal antibody BABB75, reactive with *B. bovis* RAP-1 (red fluorescence) (12). The immunofluorescence results demonstrated reactivity of the rabbit anti-15-mer motif antibody with an antigen expressed in permeabilized infected erythrocytes (green fluorescence) (**Figure 4A**) and with the surface of non-permeabilized *B. bovis* extra-cellular merozoites (green fluorescence) (**Figure 4B**). These immunofluorescence images also suggest co-localization of both antigens (orange/yellowish fluorescence) in most intracellular merozoites. A more detailed view of the pattern of reactivity of the monoclonal anti-RAP-1 antibody BABB75 (red fluorescence), and anti 15-mer RRA peptide (green fluorescence) rabbit antibodies with permeabilized infected erythrocytes is shown in panels 11-15 of **Figure 4A**, depicting a merozoite invading an erythrocyte. The RAP-1 protein, recognized by the mAb BABB75 (red fluorescence), is clearly expressed in the apical end of the parasite during erythrocyte invasion, however the reactivity of anti-RAP-1 and the anti-15-mer antibodies also overlap in the apical end (orange/yellowish fluorescence), also confirming the co-expression of both molecules and a possible role of the neutralization-sensitive RRA (22), during erythrocyte invasion.

The analysis performed on non-permeabilized extracellular merozoites is shown in **Figure 4B** where anti-RAP-1 and anti-RRA 15-mer antibodies reacted with the surface of aggregated extracellular *B. bovis* merozoites. Overall, the data supports the expression of merozoite surface exposed epitopes that are recognized by the antibodies against the 15-mer motif peptide, leading to the notion that the predicted exposed amino acids of the 15-mer motif could be a part of a merozoite surface exposed B-cell epitope.

We then examined whether B-cell epitopes in the conserved 15-mer region of RRA are also recognized by antibodies in cattle protected from *B. bovis* infection. To this end, we analyzed IgM ad IgG responses in sera derived from calves and adult cattle that were vaccinated with *in vitro* culture attenuated parasites, and then challenged with a virulent strain of *B. bovis* in previous studies (30, 31). We compared antibody responses against a synthetic peptide representing the sequence of the 15-mer motif of the *B. bovis* RRA, and a peptide representing an immunodominant B-cell epitope expressed in the CT end of RAP-1, recognized by monoclonal antibody BABB75 (12). Antibodies in the calf sera were examined at different dates following immunization with an attenuated live vaccine and challenge with virulent *B. bovis* parasites (30, 31).

**Figure 5:**
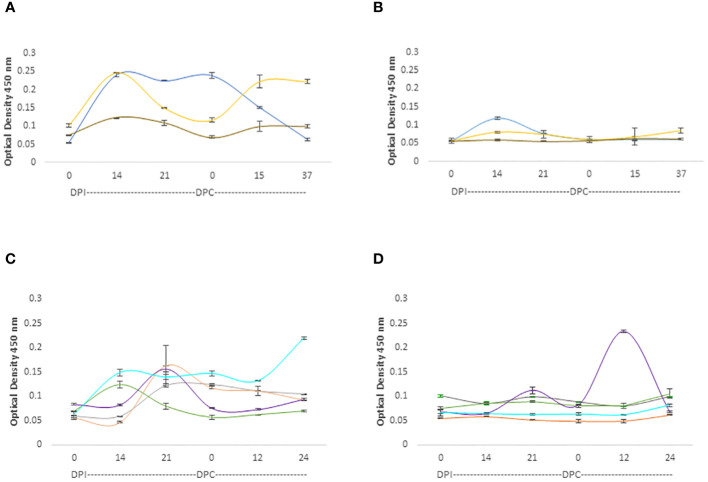
iELISA quantification of IgM antibodies against the 15-mer motif of RRA peptide and RAP-1CT B-cell epitope peptide in cattle experimentally infected and challenged with attenuated and virulent (*B*) *bovis* respectively. DPI: days post-infection with the (*B*) *bovis* attenuated strain Att- S74-T3Bo. DPC represents days post challenge with the (*B*) *bovis* strain Vir-S74-T3Bo. **(A)** IgM levels in calves against the RAP-1CT peptide; **(B)** IgM levels in calves against the RRA 15-mer peptide. **(C)** IgM levels in adult cattle against the RAP-1CT peptide; **(D)** IgM levels in adult cattle against the RRA 15-mer peptide. The IDs of the calves tested is: blue C1735, yellow C1736 brown C1738. The IDs of the adult cattle tested is: grey C1703, orange C1705, green C1706, purple C1707 and cyan C11325.

The IgM responses are represented in **Figure 5**. While both adult and young cattle mounted responses against the RAP-1CT and the RRA 15-mer peptide, the IgM responses are much higher for the 3 calves analyzed hereby (p< 0.05). (**Figure 5A**). These calves also mounted IgM responses recognizing the RRA 15-mer peptide, specially, calf C1735 (blue line, **Figure 5B**) but at much lower levels compared to the response against the immunodominant RAP-1CT peptide (p < 0.05). Regarding the IgM responses of adult cattle, although both groups generated IgM responses, there is an interesting clear and anamnestic response against the RRA NT 15-mer peptide by cow C1707 (Purple line **Figure 5D**).

**Figure 6:**
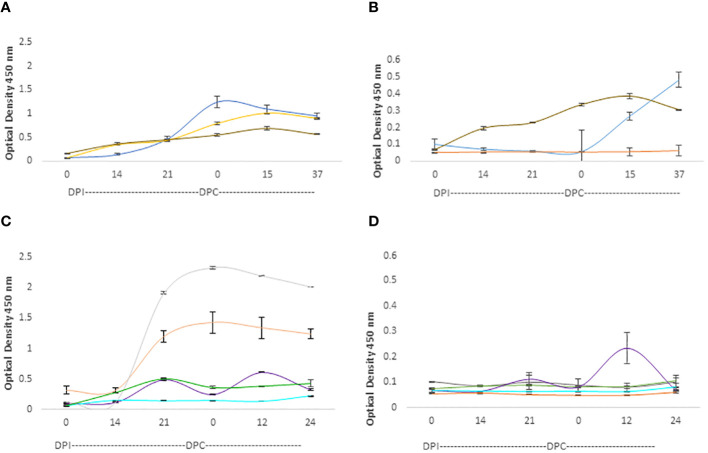
iELISA quantification of IgG antibodies against the 15-mer motif of RRA peptide and RAP-1CT B-cell epitope peptide in cattle experimentally infected and challenged with attenuated and virulent *(B) bovis* respectively. DPI: days post-infection with the (*B*) *bovis* attenuated strain Att- S74-T3Bo. DPC represents days post challenge with the (*B*) *bovis* strain Vir-S74-T3Bo. **(A)** IgG levels in calves against the RAP-1CT peptide; **(B)** IgG levels in calves against the RRA 15-mer peptide. **(C)** IgG levels in adult cattle against the RAP-1CT peptide; **(D)** IgG levels in adult cattle against the RRA 15-mer peptide. The IDs of the calves tested is blue C1735, yellow C1736 brown C1738. The IDs of the adult cattle tested is: grey C1703, orange C1705, green C1706, purple C1707 and cyan C11325.

The IgG responses in calves and adult cattle are represented in **Figure 6**. Both calves and adult cows mounted strong IgG responses against the RAP-1CT peptides, but the responses against this peptide are more consistent and uniform among calves. All three analyzed calves showed strong IgG responses against the RAP-CT peptide, and at least two calves, C1735, and C1738 (blue and brown lines, respectively, in **Figure 6B**), showed significant, albeit lower, IgG responses to the 15-mer RRA NT peptide (p <0.05). Two adult cows (C1703 and C1705 represented with grey and orange lines respectively, in **Figure 6C**) mounted strong IgG responses against the RAP-1CT peptide. Interestingly, and similar to what was found for the IgM responses, there was a strong anamnestic response for cow C1707 against the peptide representing the conserved RRA 15-mer peptide (**Figure 6D**).

Altogether, the iELISA data demonstrated the presence of at least one B-cell epitope that is recognized by antibodies in *B. bovis* infected adult and young cattle. Regardless of the quantitative differences found, we concluded from these experiments that at least one B-cell epitope is present in the 15-mer region of RRA. However, it must be noted that the magnitude of the humoral immune responses against Rap-1 CT, a known immunodominant epitope, are much higher than the responses against the epitope in the RRA 15-mer, indicating that the B-cell epitope in the RRA conserved 15-mer region is likely sub-immunodominant.

## 4 Discussion

The data obtained in this study expands our understanding of possible RAP-1 and RRA roles and provides a possible candidate well-conserved peptide for the development of peptide or other composite genes vaccines. While live vaccines remain the most effective approach to prevent acute clinical bovine babesiosis caused by *B. bovis* and *B. bigemina* (1, 30, 31), they have multiple limitations and are not available in all endemic or potentially endemic areas worldwide. In this context, subunit vaccines that can replace current live vaccines remain an attractive, but elusive goal. Several previous attempts to develop subunit vaccines based on recombinant proteins have so far failed or remain unable to provide long lasting and effective immunity against *B. bovis* (19, 20, 34–36). In addition to recombinant proteins, recent research also explored the possibility of developing subunit vaccines based on peptides including B and T lymphocyte epitopes, alone or in combination (37–41). Also, an appealing alternative version of this approach is the generation of artificial genes encoding for multiple protective B and T cell epitopes. This approach towards vaccine development can also aid in the development of new generation RNA or DNA-based vaccines. Although several epitopes that can elicit antibodies that were shown to be able to block invasion of erythrocytes by the parasite have been so far identified (39, 40), there is a shortage of information on additional protective epitopes and regions of key parasite proteins that can be targeted by peptide-based subunit vaccines. Ideally, such subunit vaccines based on peptides or representing linked protective T-and B-cell epitopes, can be used, or assembled for vaccine development. However, functionally relevant, and essential parasite molecules are also likely associated with poor or low antigenicity. This can be the case for RRA, which was shown to be poorly expressed and sub immune-dominant, but neutralization sensitive (22). Interestingly, RRA contains the typical NT region characteristic of the more immunodominant RAP-1 proteins, raising the possibility that this protein is a functional equivalent of RAP-1 that can be used by the parasite as a back up to cover for RAP-1 functions in the presence of strong RAP-1 neutralizing immune responses. In this scenario, it is possible that a RAP-1 superfamily-based vaccine should require the inclusion of both, RAP-1 and RRA recombinant proteins or protective RAP-1 and RRA B- and T- cell epitopes, in order to increase the chances to block an essential function of the parasite. Hereby, we focused on a 15-amino acid conserved region of RRA, the 15-mer, that contains a sub-immunodominant and surface exposed B-cell epitope.

Sequencing data demonstrated high levels of conservation of the 15-mer motif among RRAs from distinct species of Babesia, and full conservation among the RRAs of distinct geographic strains. Therefore, it is possible to speculate that the conservation of this motif is not a random event, but the result of natural selection due to, still unknown, functional or structural constraints. Consistent with this notion, the role of the motif may either be structural, by providing specific characteristics that will eventually influence the shape of the molecules, or it may contain residues that are critical for specific functions, which may be enzymatic activities or involvement in the interaction of RRA with other molecules. Considering its conspicuous presence and high degree of conservation among the piroplasmid RAP-1 proteins, the 15-mer motif might be an essential component of the RAP-1 and RRA molecules, which requires further characterization.

Interestingly, we found that RRA 15-mer motif-related sequences are also present in type-10 adenylate cyclases as well as in several other proteins containing ATP binding sites, such as dyneins, which are motor proteins that drive motility and microtubule-binding mechanisms that can be related to the active erythrocyte invasion by the parasite that requires rhoptry proteins (4, 6). Although the motif seems not to be located in an area with known assigned function in the type-10 adenylate cyclases, it is still rational to hypothesize a possible structural or functional role of RRA that might be related to ATP binding that may be required for parasite motility and invasion. In addition, the conservation of 15-mer motif-like regions in these molecules may be either a result of a common evolutionary origin or from convergent evolution mechanisms. Furthermore, *in silico* generated surface structural predictions also support the existence of possible relationships among ATP-biding proteins and the Babesial RRAs. Altogether, these findings may provide a rationale for developing further experiments aimed at demonstrating the possible interactions of the *B. bovis* RRA with ATP, NTPs or other ATP-binding partner molecules.

The current study revealed that the *B. bovis* RRA contains a B-cell epitope which might be expressed, at least in part, on the surface of the RRA molecules. This is supported by the Alphafold structural predictions, and the finding of surface expression of a B-cell epitope recognized by rabbit antibodies against a synthetic peptide representing the sequence of the 15-mer by immunofluorescence analysis. The use of permeabilized and nonpermeabilized parasites in this analysis is an approach that was successfully used before for the demonstration of other merozoite surface expressed molecules (32). More significantly, a B-cell epitope in the synthetic peptide representing the conserved 15-mer RRA motif was also recognized, albeit relatively weakly, by antibodies from cattle that was vaccinated with attenuated *B. bovis* parasites which survived further challenge with virulent parasites, as demonstrated in previous studies (30, 31). An anamnestic, albeit low level, IgG response is evident upon challenge of at least one vaccinated and protected calf. Overall, this is consistent with the previous characterization of RRA as an immune-subdominant antigen (22) and with the high levels of conservation of the 15-mer of RRA among *Babesia* parasites, since poorly antigenic well conserved and functionally essential regions should, at least in theory, be better suited to resist or avoid the strong selective pressures of the immune responses of the vertebrate hosts. However, it is possible, but remains to be demonstrated, that antibodies against the 15-mer motif may play a role in the protective immune response against the parasite. Thus, it is possible to speculate that the 15-mer-reacting antibodies might be needed to limit the growth of the parasite in infected animals, and to facilitate its transition to persistent infection. These thus may favor both the surviving host and the chances for transmission of the parasites via tick vectors.

In summary, bioinformatics, structural, and experimental data on a conserved 15-mer motif in this study generated information that can provide clues on the functional and immunological relevance of RRA, an understudied molecule of *B. bovis* that may be a candidate for inclusion on future recombinant vaccines. Findings of this study support the notion that the *B. bovis* RRA 15-mer motif contains a well conserved B-cell epitope that is at least partially exposed in the surface of the parasite which can generate anamnestic immune responses in *B. bovis* infected calves. It remains to be demonstrated whether the antibody responses against the RAP-1CT and RRA 15-mer B-cell epitopes play a role in protection against challenge with a virulent strain upon vaccination. These findings can also be exploited for developing functional hypothesis on the role of the members of the Babesia RRA and RAP-1 superfamily, and for the development of novel RRA/RAP-1 based peptide or multi-epitope subunit vaccines.

## Data availability statement

The original contributions presented in the study are included in the article/**Supplementary Material**. Further inquiries can be directed to the corresponding author.

## Ethics statement

The animal study was approved by Institutional Animal Care and Use Committee, University of Idaho (Protocol IACUC- #2013-66). The study was conducted in accordance with the local legislation and institutional requirements.

## Author contributions

MR: Conceptualization, Data curation, Formal analysis, Investigation, Methodology, Software, Validation, Visualization, Writing – original draft, Writing – review & editing. RB: Conceptualization, Investigation, Methodology, Writing – review & editing. JN: Data curation, Investigation, Methodology, Validation, Writing – review & editing. JL: Conceptualization, Data curation, Investigation, Methodology, Writing – review & editing. PL: Investigation, Methodology, Writing – review & editing. CS: Conceptualization, Data curation, Formal analysis, Funding acquisition, Investigation, Methodology, Project administration, Resources, Software, Supervision, Visualization, Writing – original draft, Writing – review & editing.

## Supplementary material

Supplementary Figure 1 Alignment of amino acid sequences of the RRA proteins among distinct geographical *B. bovis* strains: Missouri Mo7, Texas S79, Australian, and Argentinian strains. The sequence representing the RRA 15-mer is in red font.

Supplementary Figure 2 CLUSTAL generated alignment of amino acid sequences of the NT region (aminoacids 1-310) of RAP-1 and the RRA of *B. bovis* (62% identity). The amino acid residues that are a part of the conserved 14-15 amino acid motifs are shown in blue and red font.

Supplementary Figure 3 CLUSTAL generated alignment of the full sizes RRA and the CT end of AC1 (adenylate cyclase type 10, *Lasius niger*; GenBank accession: KMQ94175). The red box marks the area containing the conserved 15-mer of RRA.

Supplementary Figure 4 CLUSTAL generated alignment of the full sizes RRA and the CT ends of AC1 (adenylate cyclase type 10, Lasius niger; GenBank accession number: KMQ94175) and AC2: adenylate cyclase type 10-like [Nylanderia fulva]. GeneBank Accession: XP_029155514. The red box marks the area containing the conserved 15-mer of RRA.

Supplementary Figure 5 Specificity of the rabbit antibody against the 15mer of *B. bovis* RRA: **(A)**. Serological evaluation of pre-immune sera and sera from rabbits immunized with a KLH-conjugated 15mer synthetic peptide representing the conserved 15mer of *B. bovis* RRA. using iELISA using plates coated with non-KLH conjugated 15mer purified peptide (RRA NT *B. bovis*, green columns, and a random peptide of identical amino acid composition (yellow columns). **(B)**. Immunoblot analysis: Rabbit antibodies against the 15mer of *B. bovis* RRA recognized recombinant RRA (rRRA), but not the highly related recombinant RAP-1NT (rRAP-1NT) in Western blot (Panel labeled Anti-15mer RRA ab)s. A control anti-HIS antibody was used to demonstrate the presence of rRRA and rRAP-1NT proteins in the gels (Panel labeled Ant-HIS ab).
