# Supplementary material for: A conserved motif in the immune-subdominant RAP-1 related antigen of Babesia bovis contains a B-cell epitope recognized by antibodies from protected cattle
Source: Front Immunol. 2024 Apr 24;15:1380660. doi: 10.3389/fimmu.2024.1380660 (PMC11076753; doi:10.3389/fimmu.2024.1380660)
Supplement: Supplementary Figure 1 — Alignment of amino acid sequences of the RRA proteins among distinct geographical B. bovis strains: Missouri Mo7, Texas S79, Australian, and Argentinian strains. The sequence representing the RRA 15-mer is in red font. [file Presentation_1.pptx]

## Slide 1
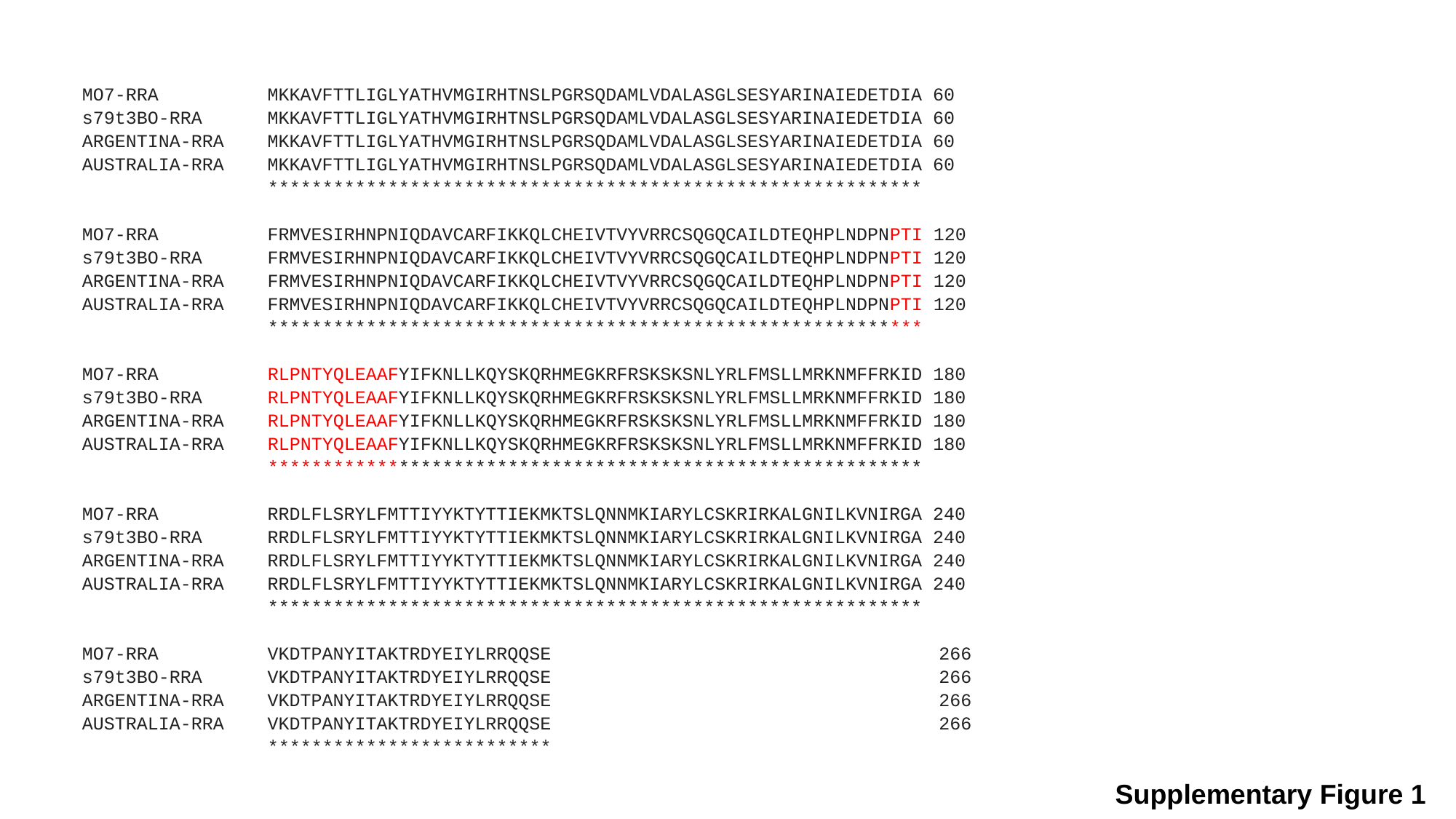

MO7-RRA MKKAVFTTLIGLYATHVMGIRHTNSLPGRSQDAMLVDALASGLSESYARINAIEDETDIA 60
s79t3BO-RRA MKKAVFTTLIGLYATHVMGIRHTNSLPGRSQDAMLVDALASGLSESYARINAIEDETDIA 60
ARGENTINA-RRA MKKAVFTTLIGLYATHVMGIRHTNSLPGRSQDAMLVDALASGLSESYARINAIEDETDIA 60
AUSTRALIA-RRA MKKAVFTTLIGLYATHVMGIRHTNSLPGRSQDAMLVDALASGLSESYARINAIEDETDIA 60
 ************************************************************
MO7-RRA FRMVESIRHNPNIQDAVCARFIKKQLCHEIVTVYVRRCSQGQCAILDTEQHPLNDPNPTI 120
s79t3BO-RRA FRMVESIRHNPNIQDAVCARFIKKQLCHEIVTVYVRRCSQGQCAILDTEQHPLNDPNPTI 120
ARGENTINA-RRA FRMVESIRHNPNIQDAVCARFIKKQLCHEIVTVYVRRCSQGQCAILDTEQHPLNDPNPTI 120
AUSTRALIA-RRA FRMVESIRHNPNIQDAVCARFIKKQLCHEIVTVYVRRCSQGQCAILDTEQHPLNDPNPTI 120
 ************************************************************
MO7-RRA RLPNTYQLEAAFYIFKNLLKQYSKQRHMEGKRFRSKSKSNLYRLFMSLLMRKNMFFRKID 180
s79t3BO-RRA RLPNTYQLEAAFYIFKNLLKQYSKQRHMEGKRFRSKSKSNLYRLFMSLLMRKNMFFRKID 180
ARGENTINA-RRA RLPNTYQLEAAFYIFKNLLKQYSKQRHMEGKRFRSKSKSNLYRLFMSLLMRKNMFFRKID 180
AUSTRALIA-RRA RLPNTYQLEAAFYIFKNLLKQYSKQRHMEGKRFRSKSKSNLYRLFMSLLMRKNMFFRKID 180
 ************************************************************
MO7-RRA RRDLFLSRYLFMTTIYYKTYTTIEKMKTSLQNNMKIARYLCSKRIRKALGNILKVNIRGA 240
s79t3BO-RRA RRDLFLSRYLFMTTIYYKTYTTIEKMKTSLQNNMKIARYLCSKRIRKALGNILKVNIRGA 240
ARGENTINA-RRA RRDLFLSRYLFMTTIYYKTYTTIEKMKTSLQNNMKIARYLCSKRIRKALGNILKVNIRGA 240
AUSTRALIA-RRA RRDLFLSRYLFMTTIYYKTYTTIEKMKTSLQNNMKIARYLCSKRIRKALGNILKVNIRGA 240
 ************************************************************
MO7-RRA VKDTPANYITAKTRDYEIYLRRQQSE	 266
s79t3BO-RRA VKDTPANYITAKTRDYEIYLRRQQSE	 266
ARGENTINA-RRA VKDTPANYITAKTRDYEIYLRRQQSE	 266
AUSTRALIA-RRA VKDTPANYITAKTRDYEIYLRRQQSE	 266
 **************************
Supplementary Figure 1

## Slide 2
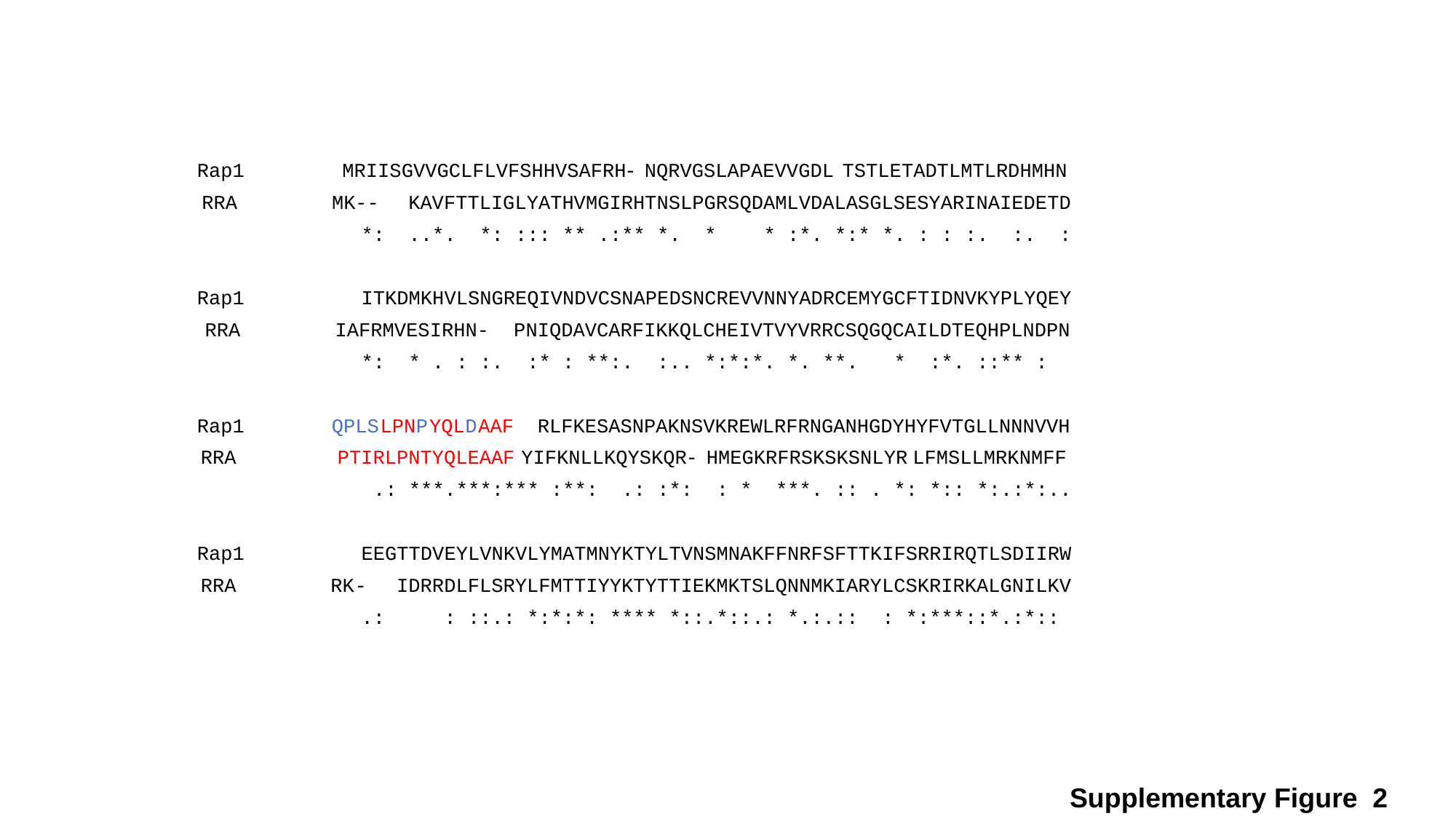

Rap1
MRIISGVVGCLFLVFSHHVSAFRH
-
NQRVGSLAPAEVVGDL
TSTLETADTLMTLRDHMHN
RRA MK
--
KAVFTTLIGLYATHVMGIRHTNSLPGRSQDAMLVDALASGLSESYARINAIEDETD
*: ..*. *: ::: ** .:** *. * * :*. *:* *. : : :. :. :
Rap1
ITKDMKHVLSNGREQIVNDVCSNAPEDSNCREVVNNYADRCEMYGCFTIDNVKYPLYQEY
RRA IAFRMVES
IRHN
-
PNIQDAVCARFIKKQLCHEIVTVYVRRCSQGQCAILDTEQHPLNDPN
*: * . : :. :* : **:. :.. *:*:*. *. **. * :*. ::** :
Rap1
QPLS
LPN
P
YQL
D
AAF
RLFKESASNPAKNSVKREWLRFRNGANHGDYHYFVTGLLNNNVVH
RRA
PTIRLPNTYQLEAAF
YIFKNLLKQYSKQR
-
HMEGKRFRSKSKSNLYR
LFMSLLMRKNMFF
.: ***.***:*** :**: .: :*: : * ***. :: . *: *:: *:.:*:..
Rap1
EEGTTDVEYLVNKVLYMATMNYKTYLTVNSMNAKFFNRFSFTTKIFSRRIRQTLSDIIRW
RRA
RK
-
IDRRDLFLSRYLFMTTIYYKTYTTIEKMKTSLQNNMKIARYLCSKRIRKALGNILKV
.: : ::.: *:*:*: **** *::.*::.: *.:.:: : *:***::*.:*::
Supplementary Figure 2

## Slide 3
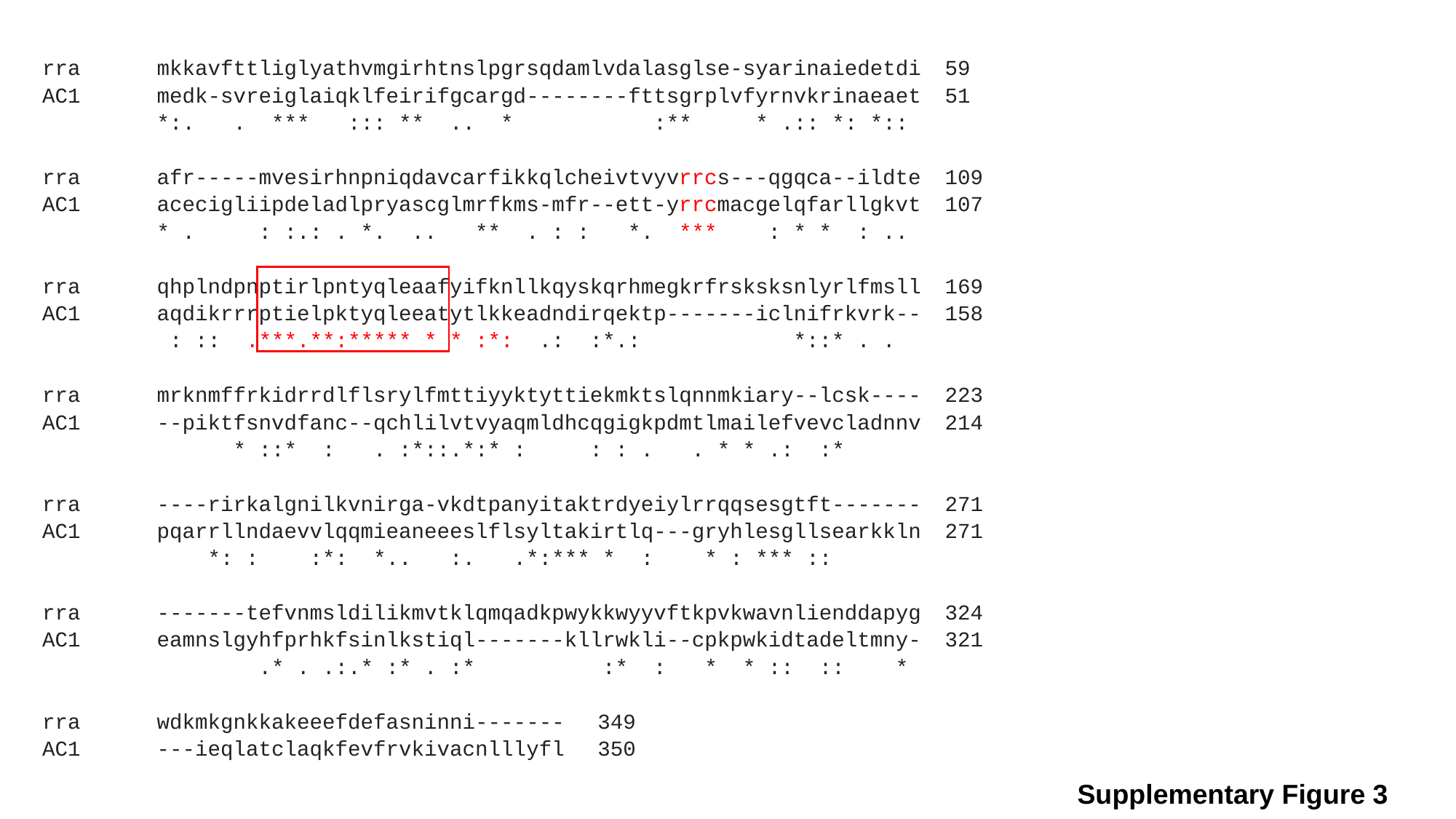

rra mkkavfttliglyathvmgirhtnslpgrsqdamlvdalasglse-syarinaiedetdi	59
AC1 medk-svreiglaiqklfeirifgcargd--------fttsgrplvfyrnvkrinaeaet	51
 *:. . *** ::: ** .. * :** * .:: *: *::
rra afr-----mvesirhnpniqdavcarfikkqlcheivtvyvrrcs---qgqca--ildte	109
AC1 acecigliipdeladlpryascglmrfkms-mfr--ett-yrrcmacgelqfarllgkvt	107
 * . : :.: . *. .. ** . : : *. *** : * * : ..
rra qhplndpnptirlpntyqleaafyifknllkqyskqrhmegkrfrsksksnlyrlfmsll	169
AC1 aqdikrrrptielpktyqleeatytlkkeadndirqektp-------iclnifrkvrk--	158
 : :: .***.**:***** * * :*: .: :*.: *::* . .
rra mrknmffrkidrrdlflsrylfmttiyyktyttiekmktslqnnmkiary--lcsk----	223
AC1 --piktfsnvdfanc--qchlilvtvyaqmldhcqgigkpdmtlmailefvevcladnnv	214
 * ::* : . :*::.*:* : : : . . * * .: :*
rra ----rirkalgnilkvnirga-vkdtpanyitaktrdyeiylrrqqsesgtft-------	271
AC1 pqarrllndaevvlqqmieaneeeslflsyltakirtlq---gryhlesgllsearkkln	271
 *: : :*: *.. :. .*:*** * : * : *** ::
rra -------tefvnmsldilikmvtklqmqadkpwykkwyyvftkpvkwavnlienddapyg	324
AC1 eamnslgyhfprhkfsinlkstiql-------kllrwkli--cpkpwkidtadeltmny-	321
 .* . .:.* :* . :* :* : * * :: :: *
rra wdkmkgnkkakeeefdefasninni-------	349
AC1 ---ieqlatclaqkfevfrvkivacnlllyfl	350
Supplementary Figure 3

## Slide 4
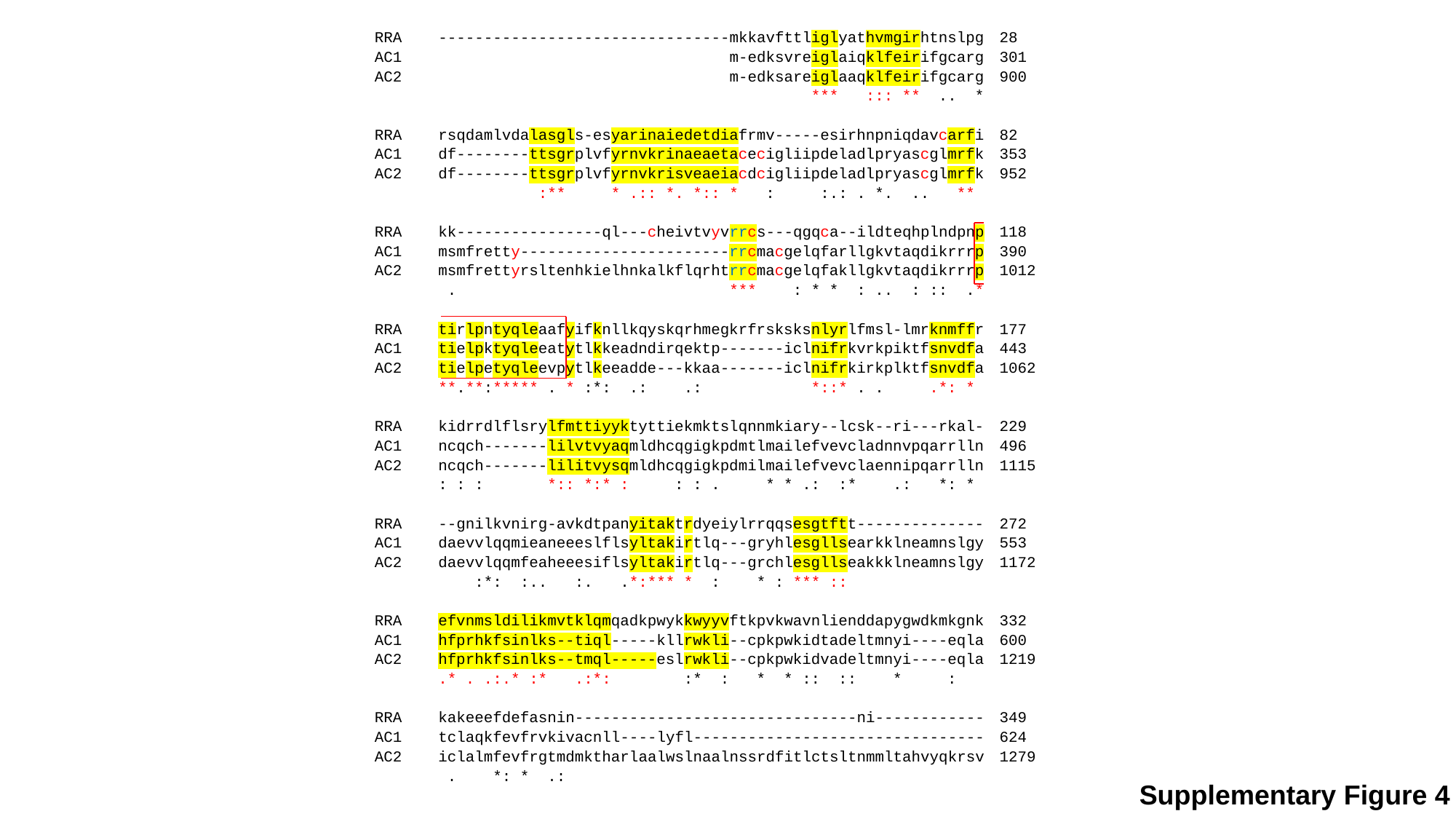

RRA --------------------------------mkkavfttliglyathvmgirhtnslpg	28
AC1 m-edksvreiglaiqklfeirifgcarg	301
AC2 m-edksareiglaaqklfeirifgcarg	900
 *** ::: ** .. *
RRA rsqdamlvdalasgls-esyarinaiedetdiafrmv-----esirhnpniqdavcarfi	82
AC1 df--------ttsgrplvfyrnvkrinaeaetacecigliipdeladlpryascglmrfk	353
AC2 df--------ttsgrplvfyrnvkrisveaeiacdcigliipdeladlpryascglmrfk	952
 :** * .:: *. *:: * : :.: . *. .. **
RRA kk----------------ql---cheivtvyvrrcs---qgqca--ildteqhplndpnp	118
AC1 msmfretty-----------------------rrcmacgelqfarllgkvtaqdikrrrp	390
AC2 msmfrettyrsltenhkielhnkalkflqrhtrrcmacgelqfakllgkvtaqdikrrrp	1012
 . *** : * * : .. : :: .*
RRA tirlpntyqleaafyifknllkqyskqrhmegkrfrsksksnlyrlfmsl-lmrknmffr	177
AC1 tielpktyqleeatytlkkeadndirqektp-------iclnifrkvrkpiktfsnvdfa	443
AC2 tielpetyqleevpytlkeeadde---kkaa-------iclnifrkirkplktfsnvdfa	1062
 **.**:***** . * :*: .: .: *::* . . .*: *
RRA kidrrdlflsrylfmttiyyktyttiekmktslqnnmkiary--lcsk--ri---rkal-	229
AC1 ncqch-------lilvtvyaqmldhcqgigkpdmtlmailefvevcladnnvpqarrlln	496
AC2 ncqch-------lilitvysqmldhcqgigkpdmilmailefvevclaennipqarrlln	1115
 : : : *:: *:* : : : . * * .: :* .: *: *
RRA --gnilkvnirg-avkdtpanyitaktrdyeiylrrqqsesgtftt--------------	272
AC1 daevvlqqmieaneeeslflsyltakirtlq---gryhlesgllsearkklneamnslgy	553
AC2 daevvlqqmfeaheeesiflsyltakirtlq---grchlesgllseakkklneamnslgy	1172
 :*: :.. :. .*:*** * : * : *** ::
RRA efvnmsldilikmvtklqmqadkpwykkwyyvftkpvkwavnlienddapygwdkmkgnk	332
AC1 hfprhkfsinlks--tiql-----kllrwkli--cpkpwkidtadeltmnyi----eqla	600
AC2 hfprhkfsinlks--tmql-----eslrwkli--cpkpwkidvadeltmnyi----eqla	1219
 .* . .:.* :* .:*: :* : * * :: :: * :
RRA kakeeefdefasnin-------------------------------ni------------	349
AC1 tclaqkfevfrvkivacnll----lyfl--------------------------------	624
AC2 iclalmfevfrgtmdmktharlaalwslnaalnssrdfitlctsltnmmltahvyqkrsv	1279
 . *: * .:
Supplementary Figure 4

## Slide 5
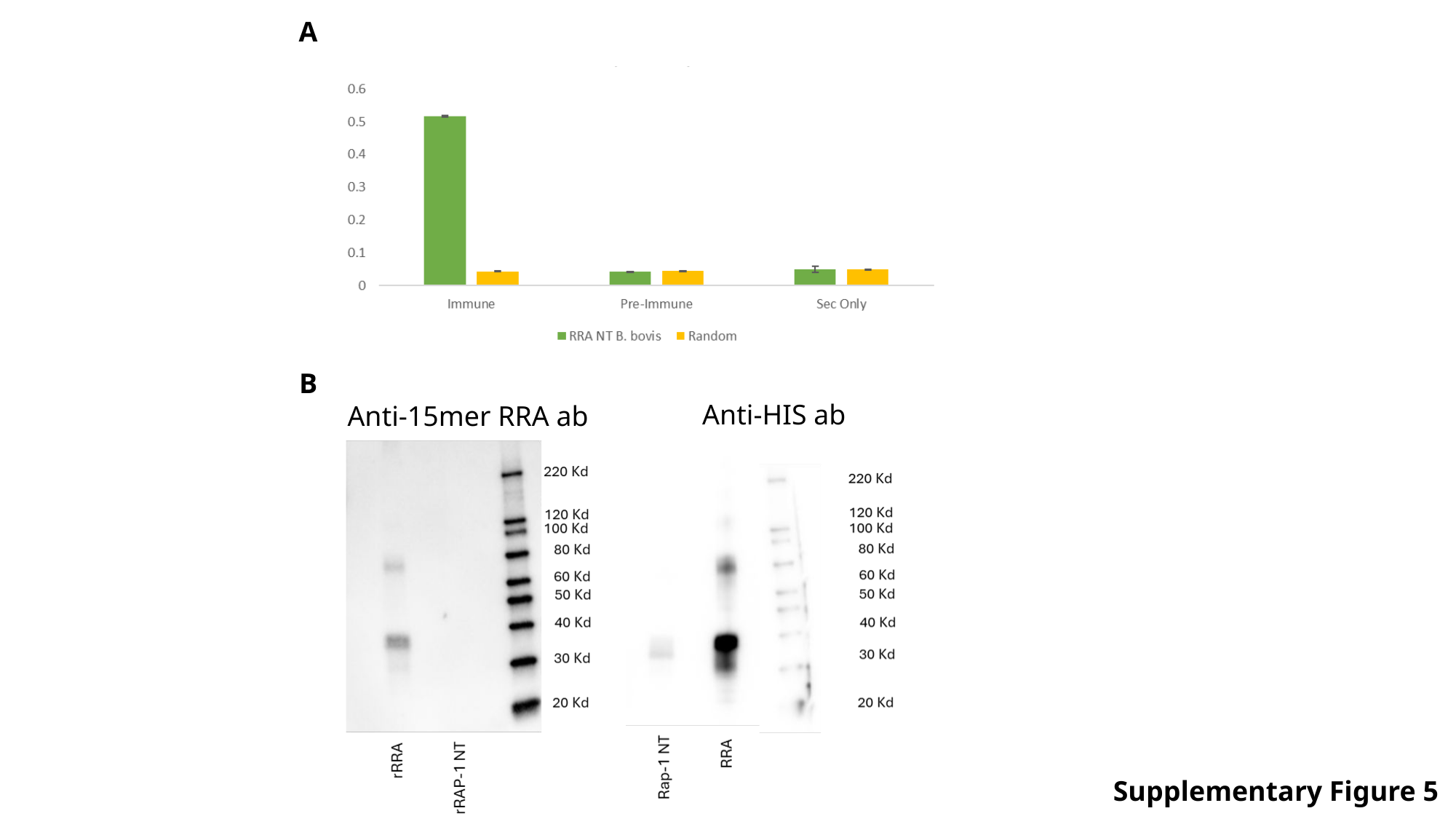

A
B
Anti-HIS ab
Anti-15mer RRA ab
Supplementary Figure 5
